# Suicidality in women with Premenstrual Dysphoric Disorder: a systematic literature review

**DOI:** 10.1007/s00737-020-01054-8

**Published:** 2020-09-16

**Authors:** E. Osborn, J. Brooks, P. M. S. O’Brien, A. Wittkowski

**Affiliations:** 1 Faculty of Biology, Medicine and Health, School of Health Sciences, Division of Psychology and Mental Health, Manchester Academic Health Science Centre, The University of Manchester, 2nd floor Zochonis Building, Brunswick Street, Manchester, M13 9PL UK; 2 Greater Manchester Mental Health NHS Foundation Trust, Manchester, UK; 3 Emeritus Professor, School of Medicine, Keele University, Staffordshire, UK

**Keywords:** Suicide, Literature review, Premenstrual syndrome, Menstrual cycle, Women

## Abstract

Previous research has identified how menstruation is an important factor in both attempted and completed suicides for women. The purpose of this review was to outline (a) the risk profile for suicidality in women who were identified to experience Premenstrual Dysphoric Disorder (PMDD), a condition characterized by severe physical and psychological changes that occur during the luteal menstrual phase, and (b) the implications of these findings for clinical practice. A systematic literature review was conducted using five databases to identify any peer-reviewed articles published between 1989 and 2019. Ten papers eligible for inclusion were identified: three pertaining to *suicide cognitions*, five to *suicide attempts* and two to both *cognitions* and *attempts*. Findings showed that suicidal thoughts, ideation, plans and attempts were strongly associated with experiences of PMDD and that these findings were independent of psychiatric co-morbidities. However, women with PMDD did not present with more severe risk profiles for suicide attempts (in terms of frequency, impulsivity and lethality) or make more frequent attempts during the luteal menstrual phase compared with suicide attempters without PMDD. Women with PMDD should be considered a high risk group for suicidality; thus, identifying and treating symptoms are vital in reducing suicide attempts. Implications for clinical practice are outlined in the discussion.

## Introduction

Nearly 800,000 people die by suicide each year, a statistic that equates to one life being lost every 40 seconds. For each person who completes suicide, it is estimated that 20 others will have made attempts, resulting in a potential figure of 16 million people worldwide (World Health Organization 2019). Thus, suicide constitutes a major public health problem for which understanding and preventing deaths have been identified as both local and global priorities (Department of Health and Social Care 2012; World Health Organisation 2014 ). With statistics consistently showing that women make more frequent attempts to end their own lives than men (Vijayakumar 2015), it is imperative that we further understand the reasons underlying suicidal behaviours in women, in order to inform future prevention and treatment.

The factors that drive an individual to suicide are often complex and varied; however, there is an increasing body of evidence that menstruation may play a significant role for some women. Studies have identified how suicide attempts in women are positively associated with particular menstrual cycle phases (Jang and Elfenbein 2019; Papadopoulou et al. 2019; Zengin et al. 2015) and with fluctuating levels of the female sex hormones oestrogen and progesterone (Mousavi et al. 2014; Baca-Garcia et al. 2010). In addition to findings for attempted suicide, several autopsy studies have also identified significantly increased incidences of completed suicides during both the luteal and menses phases (Leenaars et al. 2009; Dogra et al. 2007), leading researchers to conclude that menstrual-related changes are an important mediating factor.

Mild changes in mood are commonly reported by women prior to the onset of menses, with estimates that four in ten women experience symptoms of premenstrual syndrome (PMS; Green et al. 2017), also known as premenstrual tension (PMT). Both PMS and PMT are umbrella terms for a variety of physical and emotional changes that occur in the lead up to a menstrual bleed and can include symptoms of depression and anxiety, alongside reduced confidence and feelings of irritability (Green et al. 2017). Whilst the research literature is limited, a review of 44 studies into suicide and the menstrual cycle by Saunders and Hawton (2006) identified a significant association between experiences of PMS and suicide attempts in women, based upon the findings of three papers. In particular, the researchers cited the findings of Chaturvedi et al. (1995) who identified PMS as a risk category for suicidal behaviours and concluded that these women should be considered a high suicidal risk.

For a small minority of women, the emotional changes experienced prior to the onset of menses can be extremely severe. Premenstrual Dysphoric Disorder (PMDD) is a chronic health condition affecting 3–8% of women of menstruating age (Halbreich et al. 2003) and is characterized by debilitating cognitive, affective and behavioural changes that occur during the luteal menstrual phase and typically remit following the onset of menses (Braverman 2007). Originally termed as Late Luteal Phase Dysphoric Disorder (LLPDD) due to its cyclical nature (Diagnostic and Statistical Manual of Mental Disorders; DSM-III-R; American Psychiatric Association, 1987), and sometimes referred to as “severe PMS”, PMDD was renamed in 1994 (DSM-IV; American Psychiatric Association, 1994), with diagnosis requiring a specific pattern of symptoms that are differentiated in both severity and impact from PMS, thus capturing a distinct clinical entity and a much more severely afflicted category of women (see Table 1 for DSM-5 PMDD diagnostic criteria).

**Table 1:** DSM-5 diagnostic criteria for PMDD

Timing of symptoms	
A) In the majority of menstrual cycles, at least 5 symptoms must be present in the final week before the onset of menses, start to improve within a few days after the onset of menses and become minimal or absent in the week post menses	
Symptoms	
B) One or more of the following symptoms must be present:	
1) Marked affective lability (e.g. mood swings, feeling suddenly sad or tearful or increased sensitivity to rejection)	
2) Marked irritability or anger or increased interpersonal conflicts	
3) Markedly depressed mood, feelings of hopelessness or self-deprecating thoughts	
4) Marked anxiety, tension and/or feelings of being keyed up or on edge	
C) One (or more) of the following symptoms must additionally be present to reach a total of 5 symptoms when combined with symptoms from criterion B above:	
1) Decreased interest in usual activities	
2).Subjective difficulty in concentration	
3) Lethargy, easy fatigability or marked lack of energy	
4) Marked change in appetite; overeating or specific food cravings	
5) Hypersomnia or insomnia	
6) A sense of being overwhelmed or out of control	
7) Physical symptoms such as breast tenderness or swelling; joint or muscle pain, a sensation of “bloating” or weight gain	
Severity	
A. D) The symptoms are associated with clinically significant distress or interference with work, school, usual social activities or relationships with others	
B. E) Consider Other Psychiatric Disorders. The disturbance is not merely an exacerbation of the symptoms of another disorder, such as major depressive disorder, panic disorder, persistent depressive disorder (dysthymia) or a personality disorder (although it may co-occur with any of these disorders)	
Confirmation of the disorder	
F) Criterion A should be confirmed by prospective daily ratings during at least 2 symptomatic cycles (although a provisional diagnosis may be made prior to this confirmation). Exclude other Medical Explanations	
G) The symptoms are not attributable to the physiological effects of a substance (e.g. drug abuse, medication or other treatment) or another medical condition (e.g. hyperthyroidism)	

With quality of life estimates presenting as comparable with that of other chronic illnesses (Halbreich et al. 2003; Rapkin and Winer 2009) and findings that it can take an average of 20 years for women’s symptoms to be accurately diagnosed and treated (Osborn et al. 2020), the burden for individuals living with PMDD is huge. Recent survey data indicated that 30% of women with PMDD reported attempts to end their own life (Eisenlohr-Moul et al. 2019); thus, women with this condition are likely to be a highly vulnerable population.

Previous reviews have provided an overview of what is known about PMDD, including the epidemiology and treatment options (Hantsoo and Epperson 2015; Cunningham et al. 2009; Sepede et al. 2016), alongside reviewing the potential mechanisms by which the menstrual cycle may increase suicide risk (Owens and Eisenlohr-Moul 2018). However, to date, no review has focussed specifically on reviewing the available data for experiences of suicidality in women PMDD, despite the importance of understanding the risks for this particular subgroup.

By synthesizing the available data, this review aimed to outline the risk profile for suicidality in women of menstruating age identified to experience PMDD, LLPDD or PMS that is further defined as “severe” or “extreme” and to consider how these findings compared with both the general population and women with PMS. Suicidality in this context is used broadly to refer to a spectrum of markers from suicidal thoughts, ideation and plans, through to suicide attempts.

## Method

### Search strategy

**Fig. 1:**
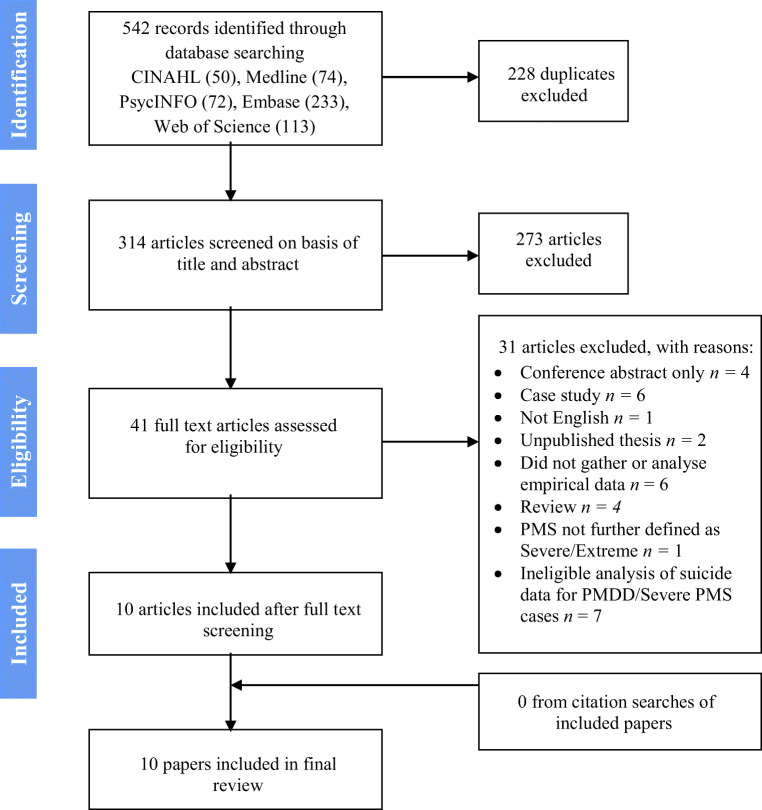
Flowchart demonstrating the literature review procedure

A systematic review of the literature was conducted using EMBASE, CINAHL, MEDLINE, PsycINFO and Web of Science on December 2, 2019. The search terms used to identify relevant papers are detailed in Table 1. Results were limited to papers published between 1989 and 2019. Figure 1, based on the Preferred Reporting Items for Systematic Reviews and Meta-Analyses (PRISMA) guidelines (Moher et al. 2009), illustrates the selection process.

### Inclusion and exclusion criteria

The inclusion criteria were (1) English language; (2) published in a peer-reviewed journal, (3) pertaining to experiences of suicidality in women of menstruating age who met the criteria for (a) PMDD, (b) LLPDD or (c) severe or extreme PMS; and (4) gathered or analysed empirical data. Reviews and single case study papers were excluded. The first 50% of studies (*n* = 157) were independently screened for inclusion by a peer (CP), which yielded a perfect rate of agreement (100%). Table 2 shows the search terms used.

**Table 2:** Search terms used in the systematic literature search

Category	Search terms
Patient/problem	Premenstrual Dysphoric Disorder OR PMDD OR Premenstrual Syndrome OR PMS or Premenstrual Tension OR PMT OR Late Luteal Phase Dysphoric Disorder OR Menstrual Cycle* OR Menstrual Disorder*
Outcome	Suicidality OR Suicidal Ideation OR Suicide OR Suicide Attempt OR Attempted Suicide OR Suicidal Thoughts OR Suicidal Behav*

### Synthesis

Key characteristics from the studies were extracted and tabulated (see Table 3). Findings were then synthesized into a discursive narrative review to provide an overview of the available data.

**Table 3:** Overview of study characteristics

Study: authors, year, location		Sample description	Study design	Assessment of PMDD	Assessment of suicidality	Data analysis	Overview of findings	Quality Rating
Suicide cognitions: Studies assessing suicidal thoughts, ideation, plans or risk (in reverse chronological order)								
1	de Carvalho et al. (2018)Brazil	727 women, aged between 21 and 32 years	Cohort: Analysis of data collected as part of a larger cross-sectional population-based study (psychosocial and biological factors in bipolar disorder: a population-based cohort of young adults)	Mini International Neuropsychiatry Interview (MINI Plus) translated into Brazilian Portuguese (Amorim 2000) The MINI assesses PMDD based upon DSM-IV criteria (American Psychiatric Association 2000)	Mini International Neuropsychiatry Interview (MINI Plus) translated into Brazilian Portuguese (Amorim 2000)	Chi-square tests were used to assess factors associated with PMDD. Poisson regression was used to obtain estimates of prevalence ratios and to adjust the analysis for potential confounders	128 women (17.6%) were identified to have PMDD Significant co-morbidity of current suicide risk and PMDD *p* = 0.002. Women with PMDD were two to three times more likely to report suicide risk than women without PMDD	59.0% Good
2	Ogebe et al. (2011) USA and Nigeria	537 adolescents, aged between 13 and 21 years	Cohort: Female adolescents attending four outpatient clinics in Lagos and Maiduguri (Nigeria) and Akron (USA) were recruited	A modified version of the Premenstrual Symptoms Screening Tool (PSST; Steiner et al. 2003) The PSST assesses PMDD based upon DSM-IV criteria (American Psychiatric Association 2000)	The Modified Mini International Neuropsychiatry Interview (Sheehan et al. 1998)	Pearson Chi-square tests were to compare the distribution of categorical variables	22 adolescents (4.1%) were identified to have PMDD 18% of adolescents with PMDD experienced co-morbid suicidal ideation, compared with 24% and 10.9% of adolescents with moderate/severe PMS and no/mild PMS, respectively	66.7% Good
3	Yonkers et al. (2003) USA	907 women, with an average age of 31 years	Cohort: Women attending six obstetric-gynaecological practices in Connecticut were invited to participate	The Premenstrual Attitudes and Perception Scale (PAPS) which was developed for this study and the Last Menstrual Period Module (LMPM) based upon the Daily Rating of the Severity of Problems (Endicott et al. 1993)	The Brief Patient Health Questionnaire (BHQ) from the Primary Care Evaluation of Mental Disorders (Spitzer et al. 2000)	Data not reported	24% of women with PMDD endorsed suicidal thoughts at any level (several days, more than half of the days, every day). 20% endorsed these thoughts for several days	38.5% Moderate
Suicide attempts: Studies assessing suicide attempts (in reverse chronological order)								
4	Shams-Alizadeh et al. (2018) Iran	120 women aged between 13 and 40 years and 120 matched controls Controls were matched with respect to age and occupational status	Control: Women admitted to the hospital following a suicide attempt and a control group of women accompanying patients on other wards were recruited	Psychiatric interview based on DSM-5 criteria for PMDD (American Psychiatric Association 2013)	Women admitted to the hospital following a suicide attempt	Pearson’s Chi-square test was used to assess the relationship between the independent variables and attempting suicide	Significantly higher frequency of PMDD in women who had attempted suicide (37/120 = 30.8%) compared with control group (6/120 = 5%); *p* = 0.001. No relationship between suicide attempts in PMDD and menstrual phase	64.1% Good
5	Ducasse et al. (2016) France	232 women aged between 18 and 53 years, with a median age of 33.8 years	Cohort: Women admitted to the hospital following a suicide attempt were recruited	Premenstrual Assessment Form Shortened version (Allen et al. 1991) using questions that met criteria from the DSM-5 (American Psychiatric Association 2013)	Women admitted to the hospital following a suicide attempt	Associations were calculated with odds ratios and 95 confidence intervals and logistic regression models	High prevalence of PMDD in women who had attempted suicide (23%: 51/232) Severity of suicide attempt was not significantly associated with PMDD. No significant difference between presence of PMDD and menstrual cycle phase of attempt	61.5% Good
6	Soydas et al (2014) Turkey	70 women with PMDD, aged between 20 and 41 years and 78 healthy controls	Control: Women with PMDD who were referred to an outpatient psychiatry service and a control group comprised of hospital staff and staff relatives were recruited	Premenstrual Syndrome Scale (PMSS; Gencdogan 2006*)*	Structured Clinical Interview for Axis I disorders (SCID; First et al. 1997), translated into Turkish (Özkürkçügil et al. 1999)	Chi-square tests were used to assess the relationships between variables	Significantly higher frequency of historical suicide attempts in women with PMDD (7.1%) compared with control group (0%); *p* = 0.024	56.4% Good
7	Baca-Garcia et al. (2004) Spain	125 women and 83 controls. Average ages were 30.6 years and 32.7 years, respectively	Control: Women admitted to the hospital following a suicide attempt and a control group of blood donors were recruited	Clinical interviews based upon DSM-IV criteria for PMDD (American Psychiatric Association 2000)	Women admitted to the hospital following a suicide attempt Impulsivity was assessed using the Beck Suicidal Intent Scale (SIS-I, Beck et al. 1974). Lethality was assessed using the Lethality Rating Scale (Beck et al. 1975)	Fisher’s exact tests were used to compare diagnosis and suicide attempt features between suicide attempters with and without PMDD. Chi-square tests were used to assess menstrual cycle phase	Significantly higher prevalence of PMDD in women attending the emergency room following a suicide attempt (54%: 67/125) compared with controls (6%: 5/83); *p* < 0.001. No significant difference regarding lethality and impulsivity of attempts No increase in suicide risk during luteal phase	43.6% Moderate
8	Wittchen et al. (2002)	1488 women, aged 14–24 years	Cohort: Analysis of data collected as part of a larger cross-sectional population-based study	PMDD module from the Munich Composite International Diagnostic Interview (M-CIDI; Wittchen and Pfister 1997) based upon DSM-IV criteria (American Psychiatric Association 2000)	Munich Composite International Diagnostic Interview (M-CIDI; Wittchen and Pfister 1997)	Odds ratio and 95% confidence intervals were calculated to compare PMDD and non-PMDD cases	112 (7.4%) women were identified to have PMDD High risk of suicide attempts among PMDD cases but only moderately increased risks of suicidal ideation. Higher prevalence of women reporting at least one suicide attempt during their lifetime (15.8%) compared with non-PMDD women (3.2%)	59.0% Good
Suicide cognitions + Suicide attempts: Studies assessing suicidal thoughts, ideation, plans or risk and suicide attempts								
9	Pilver et al. (2013) USA	3965 women, aged 18–40 years with an average age of 28.8 years	Cohort: Analysis of data collected as part of a larger cross-sectional population-based study (collaborative psychiatric epidemiology surveys CPES data set)	Premenstrual Syndrome module of the World Mental Health Composite International Diagnostic Interview (WMH-CIDI), based upon DSM-IV criteria	Statements about suicidal behaviours where positive responses were scored “1” and negative responses “0”	Three sets of logistic regression models were constructed to assess the magnitude and direction of the association between PMDD status and suicidal behaviours and to control for possible sources of confounding	PMDD was strongly and independently associated with suicidal behaviours. Women with PMDD significantly more likely than women with PMS and no premenstrual symptoms to report suicide attempts (16.2%, 7.4%, 4.9%), suicidal ideation (37.4%, 22% and 13.3%) and suicide plans (19.1%, 7.6% and 4.6%); *p* = < 0.05	74.4% Good
10	Hong et al. (2012) South Korea	2499 Korean women, aged between 18 and 49 years	Cohort: Analysis of data collected as part of a larger cross-sectional population-based study (Korean Epidemiologic Catchment Area (KECA) study)	A translated version of the PMDD module from the Composite International Diagnostic Interview (WHO-CIDI; Kessler and Üstün 2004*)* based upon DSM-IV PMDD criteria (American Psychiatric Association 2000)	Korean version of the Composite International Diagnostic Interview module on suicide (K-CIDI; Cho et al. 2002)	Odds ratios and 95% confidence intervals and logistic regression analyses were used to compare PMDD and non-PMDD cases	59 women (2.4%) were identified to have PMDD Both 12-month and lifetime prevalence of suicide ideations, plans and attempts were significantly associated with PMDD Lifetime PMDD vs non-PMDD: suicidal ideation (45.8% vs.17.3%), suicide plans (16.9% vs. 4.2%) and suicide attempts (13.6% vs. 3.9%)	79.5% Excellent

### Assessment of methodological quality and risk of bias

In order to provide an indication of the methodological bias inherent in each study, the included papers were assessed using the Quality Assessment Tool for Studies with Diverse Designs (QATSDD; Sirriyeh et al. 2012). Thirteen items relevant to quantitative studies were rated on a 4-point scale, from “not at all” (0) to “complete” (3), and percentage scores were calculated based on the actual score and the maximum total score. A random 50% of studies were assessed by an independent peer (GCY); the kappa score for the interrater reliability was 0.88, indicating an “almost perfect” level of agreement (see Table 3 for study ratings).

## Results

### Selection of studies

The search strategy identified 542 references. After removing duplicates, the titles and abstracts of 314 articles were screened for inclusion. Forty-one potentially relevant papers were identified for full-text evaluation, but 31 papers were subsequently excluded (see Fig. 1). Ten studies met inclusion criteria and were included in this review.

### Study and participant characteristics

Included articles were published between 2002 and 2018 and originated from nine different countries. No papers pertaining to LLPDD or “severe” or “extreme” PMS and suicidality were identified; thus, this review pertains solely to the current classification of PMDD. Of the ten papers included, three studies utilized a case control design, four were epidemiological cohort studies, and three were cross-sectional studies of identified patient groups (see Table 3).

Participants comprised of 10,951 women aged between 13 and 53 years, including 281 control participants (2.6%). From these women, 725 (6.6%) were identified to have PMDD (see Table 3); however, Yonkers et al. (2003) did not report the prevalence data for PMDD within their sample.

### Quality assessment

QATSDD scores for the included articles ranged between 38.5 and 79.5%, with a mean average of 60.3%. Yonkers et al. (2003) obtained the lowest score, due to limited methodological descriptions and a lack of clarity regarding several key criteria. In total, one paper was rated as having “excellent” quality, seven as “good” and two as “moderate”. As this is the first review to evaluate suicidality in women with PMDD, all studies were included in the final synthesis in order to provide a comprehensive overview of the available research.

### PMDD diagnosis

Nine studies reported that participants were assessed for PMDD based upon the diagnostic criteria outlined in the DSM, using variable methods (see Table 3 for an overview). Yonkers et al. (2003) reported using a questionnaire that was designed specifically for their study. However, as prospective charting of two symptomatic cycles is required for full DSM diagnosis, PMDD diagnoses should be considered provisional for all studies included in this review.

### Assessment of suicidality

Suicidality was assessed using structured diagnostic interviews (*n* = 5), standardized questionnaires (*n* = 1) and statements about suicide for which affirmative responses were scored (*n* = 1). In addition, three studies recruited women from a hospital following a suicide attempt (see Table 3).

### Summary of suicidality findings

Findings were grouped according to the type of suicidality being reported, including *suicide cognitions* (*n* = 5) and *suicide attempts* (*n* = 7). Of the ten studies included in this review, two studies reported data pertaining to both *cognitions* and *attempts* and therefore featured in both groupings. Supplementary data relating to PMS comparison groups, suicide risk profiles (in terms of frequency, impulsivity and severity), the influence of Axis I disorders and menstrual cycle phase were also summarized.

### Study findings

#### Suicide cognitions

In 2018 de Carvalho et al. reported findings from 727 Brazilian women, identifying that women with PMDD (*n* = 128) were two or three times more likely to report “current suicide risk” (p.45), compared with women without PMDD. Whilst the authors did not further specify the suicide features that constituted “risk”, several studies have reported data showing that women with PMDD described increased *suicide cognitions*, including suicidal thoughts, ideation and plans. First, Yonkers et al. (2003) assessed 907 women in Connecticut, USA. The researchers found that 24% of women with PMDD endorsed suicidal thoughts at any level (several days, more than half of the days or every day) and that 20% of these women endorsed suicidal thoughts for several days.

Data pertaining to suicidal ideation and plans were reported by three further studies. Both Hong et al. (2012) and Pilver et al. (2013) analysed data from large epidemiological studies based in South Korea and the USA, respectively. Together, these studies included data from 6464 women aged between 18 and 49 years, from which 227 women (3.5%) were identified to experience PMDD. The frequency of suicidal ideation and plans was analysed among PMDD and non-PMDD cases, and data were reported for both 12-month prevalence (Hong et al. 2012) and lifetime prevalence (Hong et al. 2012; Pilver et al. 2013).

In both studies, the researchers identified that women with PMDD were significantly more likely to report suicidal ideation and plans, compared with women without PMDD. Prevalence rates for lifetime suicidal ideation in women with PMDD were 45.8% and 37.4%, compared with 17.3% and 13.3% for women without PMDD, respectively (Hong et al. 2012; Pilver et al. 2013). Likewise, prevalence rates for suicide plans in women with PMDD were 16.9% and 19.1%, compared with 4.2% and 4.6% for women without PMDD, respectively (Hong et al. 2012; Pilver et al. 2013). In addition to a significant lifetime prevalence, Hong et al. (2012) also identified a significant 12-month prevalence of both suicidal ideation and plans, findings that were independent of social desirability and demographic covariates.

Furthermore, Ogebe et al. (2011) reported findings for adolescents aged between 13 and 21 years. In this study, the researchers identified that 18% of adolescents with PMDD reported suicidal ideation, in comparison with 10.9% of adolescents with no or mild PMS symptoms. In sum, women with PMDD are significantly more likely than women without PMDD to endorse *suicide cognitions* and therefore present an increased risk for suicidality. Importantly, these findings were not limited by age, social desirability or demographic covariates and were reported across a mixture of America, African and Asian cultures.

#### Suicide attempts

Data pertaining to suicide attempts were reported in seven studies and were further defined into self-report data (*n* = 4) and hospital admissions data (*n* = 3).

##### Self-report data

Wittchen et al. (2002) explored the prevalence and co-morbidities of PMDD in a community sample of 1488 young women, living in Munich, Germany. During their analysis, the authors identified a “remarkably high risk” (p.128) of suicide attempts among PMDD cases (*n* = 112), with 15.8% reporting at least one suicide attempt during their lifetime, compared with a frequency of 3.2% for women without PMDD. Since these findings, three further studies have reported similar observations.

In their epidemiological studies, Hong et al. (2012) and Pilver et al. (2013) also assessed participants for a history of suicide attempts and identified a significantly higher prevalence of previous suicide attempts of 13.6% and 16.2% respectively in women with PMDD, compared with women with no premenstrual symptoms (Hong et al. 2012: 3.9%; Pilver et al. 2013: 4.9%). Soydas et al. (2014) compared 70 Turkish women with PMDD to a healthy control group and identified a significantly higher frequency of historical suicide attempts in the PMDD group (7.1%) compared with the control group (0%). Thus, findings consistently show that women with PMDD are significantly more likely than women without PMDD to self-report previous suicide attempts.

##### Hospital admissions data

Three studies assessed the prevalence of PMDD among women who had been admitted to the hospital following a suicide attempt. In the first study, Baca-Garcia et al. (2004) assessed 125 women who were admitted to the emergency room of a Spanish general hospital following a suicide attempt and compared them with 83 female blood donor controls: 54% of the women who had attempted suicide met the diagnostic criteria for PMDD (67/125), in contrast to just 6% (5/83) of the control group. Ducasse et al. (2016) collected data from 232 women consecutively hospitalized in a French psychiatric unit following a suicide attempt and found that 23% retrospectively met the diagnostic criteria for PMDD (51/232). Finally, Shams-Alizadeh et al. (2018) studied 120 women admitted to a general hospital in Iran following a suicide attempt compared with a matched control group. In accordance with the previous findings, the researchers identified that PMDD was significantly more frequent in women who had attempted suicide (30.8%; 37/120), compared with the control group (5%; 6/120).

In summary, women with PMDD are significantly more likely to self-report previous suicide attempts, compared with women without PMDD. In addition, hospital admission studies show that a remarkably high proportion of female suicide attempters retrospectively met the criteria for PMDD.

### Comparison with PMS

Pilver et al. (2013) identified that women with PMDD were significantly more likely to report suicidal ideation (37.4%), plans (19.1%) and attempts (16.2%) compared with women with PMS (22%, 7.6% and 7.4%, respectively). Likewise, Shams-Alizadeh et al. (2018) found that PMDD was significantly more frequent in a group of female suicide attempters compared with a control group, whilst no differences were identified in the prevalence of PMS between the two groups. However, Ogebe et al. (2011) identified that 24% of adolescents with PMS reported suicidal ideation, compared with just 18% of adolescents with PMDD. Thus, whilst there is some evidence to support an increased risk profile for women with PMDD, findings to date are contradictory and require further research.

### Suicide attempt risk profiles

Data pertaining to the frequency, impulsivity and lethality of suicide attempts were reported by both Baca-Garcia et al. (2004) and Ducasse et al. (2016). Their findings showed that women with PMDD who had attempted suicide did not exhibit a more severe risk profile compared with suicide attempters without PMDD. Although Baca-Garcia et al. (2004) identified that 59% of women with PMDD had made a previous suicide attempt and that 32% had made more than two previous attempts, these findings were not significantly different to the frequencies reported by non-PMDD cases. Likewise, no significant differences were identified regarding the impulsivity of suicide attempts between women with and without PMDD in either study, or regarding the lethality of suicide attempts.

### Influence of Axis I disorders

Psychiatric co-morbidities are commonly experienced by women with PMDD; therefore, it is important to establish whether the relationship identified between PMDD and suicidality exists independently. Several studies included in this review reported data that controlled for the presence of co-morbid Axis I psychiatric disorders, with findings showing that the relationships between PMDD and suicidality largely remained significant after these adjustments were made. Specifically, Pilver et al. (2013) identified that the strong associations between PMDD and suicidal ideation, plans and attempts were independent of psychiatric co-morbidity, Wittchen et al. (2002) identified that suicide attempts in women with PMDD remained significant after controlling for major depressive disorders, and Hong et al. (2012) identified that both lifetime and 12-month suicidal ideation remained significant, when adjustments for psychiatric disorders were made, although significant associations with suicide plans and attempts disappeared.

### Relationship to menstrual cycle phase

With PMDD symptoms commencing during the week(s) prior to the onset of menses, it would be anticipated that there would be an increased frequency of suicide attempts during the luteal menstrual phase for women with PMDD. However, Baca-Garcia et al. (2004), Ducasse et al. (2016) and Shams-Alizadeh et al. (2018) all reported finding no differences in cycle phase between suicide attempters with and without a retrospective diagnosis of PMDD. Thus, despite the significant associations identified between PMDD and suicidality, women with PMDD did not seem to be more likely to attempt suicide during the luteal menstrual phase, when symptoms are expected to reach peak severity.

## Discussion

This is the first systematic review to report on the suicidal experiences of women who were identified to have Premenstrual Dysphoric Disorder (PMDD). Findings showed that suicidal thoughts, ideation, plans and attempts were strongly associated with experiences of PMDD. These findings appeared independent of psychiatric co-morbidities and were identified using a variety of methodologies. However, no significant differences were identified between PMDD and non-PMDD cases regarding suicide attempt risk profiles in terms of frequency, impulsivity or lethality or menstrual cycle phase. These findings provide further evidence to substantiate important associations between the menstrual cycle and suicidality and offer new insights into the risk profiles for women with PMDD.

Owing to the significant relationship previously identified between PMS and suicidality (Saunders and Hawton 2006), our findings of positive associations between PMDD and both suicide *cognitions* and *attempts* were expected, given that the current review focussed upon a more severely afflicted population. Likewise, findings by Pilver et al. (2013) of a significantly increased prevalence of suicidal experiences in women with PMDD, compared with women with PMS, provided further indication of the vulnerability of this particular subgroup. However, the inconsistencies in data reported by Ogebe et al. (2011) highlight how this is an area that requires further research. In contrast, findings of a lack of association between suicide attempts and the luteal menstrual phase, alongside the remarkably high prevalence of PMDD in female suicide attempters admitted to hospital, were unexpected.

With PMDD symptoms typically occurring during the week prior to the onset of menses, women with PMDD would be expected to attempt suicide more often during the luteal menstrual phase, when their symptoms reached peak severity. However, this review identified no differences in menstrual cycle phase between suicide attempters with and without PMDD. Whilst these results were surprising, they may be explained by a recent study by Osborn et al. (2020) who identified that, for some women, PMDD symptoms were experienced for up to 3 weeks prior to the onset of menses, rather than being confined solely to the week prior to menstruation, as is often cited. Thus, future research assessing the daily severity of both PMDD symptoms and suicidality, alongside the women’s menstrual phase, would be beneficial in further exploring this relationship.

Whilst no differences were identified between PMDD and non-PMDD cases with regard to the impulsivity of suicide attempts in this review, additional findings reported by Baca-Garcia et al. (2004) identified differences in personality trait level impulsivity between the two groups. Importantly, women with PMDD were found to have significantly more impulsive traits on the Barratt Impulsivity Scale (BIS; Patton et al. 1995) compared with the control group; however, this finding became non-significant following Bonferroni correction. Nonetheless, the researchers identified a possibility that PMDD is a risk factor for suicidal behaviour due to an association with impulsivity. Subsequent research has confirmed heightened levels of impulsivity with both PMDD and premenstrual symptom severity (e.g. Dawson et al. 2018; Petersen et al. 2016), making this a hypothesis that would benefit from future research. Future research should also be directed at the clinically most meaningful assessment of impulsivity in this group of women and its role as a potential risk factor alongside the role that personality traits may play. It should be noted that the majority of studies included in this review reported that women with co-existing psychiatric disorders were excluded from their samples. Finally, the influence of age on suicidality in relation to PMDD warrants further investigation.

### Limitations of this review and the included studies

The inclusion criteria for this review specified English language and peer-reviewed articles only. Whilst only one foreign language study was excluded, we acknowledge that the introduction of language and publication biases is a possible limitation of this review.

Furthermore, it is important to acknowledge that for all of the studies included in this review, diagnoses of PMDD were both provisional and retrospective. In order to meet DSM criteria for PMDD, women are required to complete prospective daily ratings of their symptoms during at least two symptomatic menstrual cycles (American Psychiatric Association, DSM-5 2013). Whilst prospective charting would have placed considerable additional demands on study participants, it would have greatly strengthened the validity of the reported data, given that women’s retrospective recall of premenstrual symptoms is considered unreliable (Dell 2004).

Notably, several studies in this review identified prevalence statistics that were substantially larger than typical population estimates for PMDD of between 3 and 8% (Halbreich et al. 2003). For example, de Carvalho et al. (2018) reported a PMDD prevalence rate of 17.6% in a sample of young Brazilian women. These findings indicate that samples may have included women who experienced the less severe symptoms of PMS and may reflect differences in the assessment methods and psychometric tools that were used by the studies. Thus, all results pertaining to provisional PMDD diagnoses in this review should be interpreted with caution.

Although significant associations were identified between PMDD and suicidal thoughts, ideations, plans and attempts, the findings of this review are unable to infer causality. Women who attempt suicide likely do so for many reasons and the data outlined here indicates that PMDD is only one factor. Whilst several studies reported controlling for the presence of psychiatric co-morbidities, we acknowledge the difficulty in achieving this, given the considerable overlap between PMDD symptoms and common mental health disorders. As previously mentioned, the majority of included studies excluded women with any co-existing psychiatric disorders. The challenge for clinicians of discerning between a treatable health condition and other severe psychiatric conditions has been discussed by Osborn et al. (2020) and, combined with the findings of this review, highlights the clinical complexities associated with this condition and the potential for devastating outcomes. Finally, the findings of this review cannot account for instances of completed suicide in women with PMDD. Whilst there are many examples of autopsy studies investigating the menstrual phase of women who have lost their lives to suicide (e.g. Leenaars et al. 2009; Dogra et al. 2007; Chetankumar and Kokatanur 2016), to date, there are no such studies looking at the associations between completed suicide and PMDD; thus, this review pertains to non-fatal suicidality only.

### Implications for clinical practice

Owing to the strong relationships identified between PMDD and suicidality in this review and the high levels of PMDD identified in female suicide attempters, there is an obligation on clinicians to consider hormonal explanations for all women who endorse suicidal thoughts, ideation, plans or attempts. In clinical practice, the premenstrual symptomology of women who report or attempt suicide should be routinely assessed using a clinical assessment and/or a brief standardized screening measure, such as the Premenstrual Symptoms Screening Tool (PSST; Steiner et al. 2003), alongside encouraging women to prospectively chart their symptoms and experiences. Upon PMDD being identified, it is imperative that appropriate support and treatments are available for women in order to reduce suicide attempts and the likelihood of subsequent unnecessary deaths.

## Conclusions

Women with PMDD should be considered a high-risk group for suicidality, including increased vulnerabilities for suicidal thoughts, ideation, plans and attempts. Detecting and treating PMDD symptoms is of paramount importance in order to reduce suicide attempts and save women’s lives; thus, the routine screening of all women who present with suicidality is encouraged.
